# Acute BDNF Treatment Upregulates GluR1-SAP97 and GluR2-GRIP1 Interactions: Implications for Sustained AMPA Receptor Expression

**DOI:** 10.1371/journal.pone.0057124

**Published:** 2013-02-27

**Authors:** Hussam Jourdi, Mohamed Kabbaj

**Affiliations:** Department of Biomedical Sciences, College of Medicine, Florida State University, Tallahassee, Florida, United States of America; Western University of Health Sciences, United States of America

## Abstract

Brain-derived neurotrophic factor (BDNF) plays several prominent roles in synaptic plasticity and in learning and memory formation. Reduced BDNF levels and altered BDNF signaling have been reported in several brain diseases and behavioral disorders, which also exhibit reduced levels of AMPAr subunits. BDNF treatment acutely regulates AMPA receptor expression and function, including synaptic AMPAr subunit trafficking, and implicates several well defined signaling molecules that are required to elicit long term potentiation and depression (LTP and LTD, respectively). Long term encoding of synaptic events, as in long term memory formation, requires AMPAr stabilization and maintenance. However, factors regulating AMPAr stabilization in neuronal cell membranes and synaptic sites are not well characterized. In this study, we examine the effects of acute BDNF treatment on levels of AMPAr-associated scaffolding proteins and on AMPAr subunit-scaffolding protein interactions. We also examine the effects of BDNF-dependent enhanced interactions between AMPAr subunits with their specific scaffolding proteins on the accumulation of both types of proteins. Our results show that acute BDNF treatment upregulates the interactions between AMPAr subunits (GluR1 and GluR2) with their scaffold proteins SAP97 and GRIP1, respectively, leading to prolonged increased accumulation of both categories of proteins, albeit with distinct mechanisms for GluR1 and GluR2. Our findings reveal a new role for BDNF in the long term maintenance of AMPA receptor subunits and associated scaffolding proteins at synapses and further support the role of BDNF as a key regulator of synaptic consolidation. These results have potential implications for recent findings implicating BDNF and AMPAr subunits in various brain diseases and behavioral disorders.

## Introduction

AMPA receptors (AMPAr) are required for many important functions in the adult and developing brain, including synaptic plasticity, and memory formation and maintenance. Binding of glutamate to AMPAr generates depolarizing currents in postsynaptic neurons that account for most of the fast excitatory neurotransmission in the mammalian brain. Interactions between specific scaffolding proteins with AMPAr subunits are important elements for the proper functioning of AMPA receptors, and in synaptic plasticity, development, and synaptic maturation. At the molecular level, AMPAr activation initiates a series of intracellular events resulting from protein-protein interactions involving specific scaffolding proteins, collectively called PDZ proteins. Among several well characterized PDZ proteins, post-synaptic density protein of 95 kDa (PSD-95) and synapse-associated protein of 97 kDa (SAP97) share homology with membrane-associated guanylate kinases (MAGUK) and primarily interact with the AMPAr subunit GluR1 [1], [2]. AMPAr subunits GluR2 and GluR3 interact with a different set of PDZ proteins comprising glutamate receptor-interacting protein1 (GRIP1), AMPAr binding protein (ABP/GRIP2), and protein interacting with C kinase 1 (Pick1) [3]. Several kinases and phosphatases exert rapid and tight control over these interactions, as they influence AMPAr trafficking to synaptic sites and their insertion into the cell membrane and removal from the synapse, and play important roles in long term potentiation (LTP) and long term depression (LTD) [2], [4]–[13]. Upregulation of AMPAr subunits at synaptic sites is important for long term encoding of synaptic events, such as in learning and in preservation of long term memory [14], [15]. However, characterization of the factors involved in AMPAr stabilization and maintenance has not received much attention.

Several brain diseases and behavioral disorders are associated with reduced neurotrophin expression and signaling and many of these ailments also exhibit lower expression levels of both AMPA receptor subunits and interacting scaffolding proteins [16]–[21]. Unfortunately however, little attention has been given to whether scaffolding proteins are contributing factors in the manifestation of diseased states in which both AMPAr levels and BDNF signaling are compromised [22], [23].

Accordingly, we set to elucidate the contributions of scaffolding proteins to BDNF’s roles in regulating AMPAr subunits because this can contribute to pathological conditions where expressions of BDNF and AMPAr subunits are compromised. In the current study, we use several strategies to elucidate the role of BDNF in the stabilization of both AMPAr subunits with their scaffolding proteins and to analyze the relevance of BDNF signaling in controlling their interactions and increasing their protein accumulation: 1) RNA interference (RNAi) in cultured neurons to knock-down GluR1 and study its impact on GluR1 and SAP97 protein levels; 2) overexpression of chimeric proteins made of enhanced green fluorescent protein fused to the C-terminal domains of GluR1 or GluR2 (EGFP-R1 and EGFP-R2, respectively) to compete with native AMPAr subunits’ binding to their scaffolding proteins and examine whether this also alters BDNF’s effects on SAP97 and GRIP1; 3) BDNF treatment of HEKTrkB cell line (HEK293 cells expressing stable levels of the BDNF receptor TrkB) to examine whether acute and prolonged BDNF treatments affect exogenous GluR2 and GRIP1 protein levels and alters their interactions and accumulation. Our findings indicate that BDNF treatment increases AMPAr protein levels, and that a divergence exists in the mechanisms regulating the accumulation of GluR1 and GluR2 by BDNF because of differential regulation of AMPAr subunit interaction with their specific scaffolding proteins.

## Materials and Methods

### Ethics Statement

All of the animal experiments described were approved by the Animal Use and Care Committee guidelines of Niigata University (Niigata, Japan) and performed in accordance with the guidelines of NIH-USA. Every effort was made to minimize the discomfort of the animals in addition to the number of animals used in the experiments.

### Neuronal Cultures

Cerebral neocortices of embryonic day (E) 18 Sprague-Dawley rats were dissociated with papain (1 mg/ml) and cells plated into Dulbecco′s-modified Eagle medium (DMEM) supplemented with 10% calf serum onto poly-D-lysine-coated chamber slides or culture dishes, at low to medium density (500–800 cells/mm^2^) in experiments used for immunostaining, western blotting and viral infection, and at higher densities (800–1200 cells/mm^2^) for RNAi transfection experiments. One h after plating, neurons were transferred to DMEM supplemented with nutrient mixture N2 (N2-DMEM), and fetal bovine serum (2% final concentration), and kept overnight in a humidified chamber at 37 °C in 5% CO_2_. Cultured neurons were then maintained in serum-free N2-DMEM medium or in neurobasal medium supplemented with nutrient mixture B27 (B27-NBM). Untreated control cultures or cultures that were supplemented with purified human recombinant BDNF (50 ng/ml; Sumitomo Chemical Ltd., Tokyo, Japan; Millipore, Temecula, CA) were used for all experiments (see below).

### Immunostaining

Cultured neurons were washed with phosphate-buffered saline and fixed for 20 min with 4% paraformaldehyde in 0.1 M phosphate buffer (pH 7.4). Neurons were immunostained with the following antibodies: anti-GluR1 N-terminus antibody (10 µg/ml; Millipore) [24], anti-GluR1 C-terminus antibody (10 µg/ml) [25], [26], anti-GluR2 N-terminus (5 µg/ml; Millipore), anti-GluR2/3 (10 µg/ml; Millipore), or anti-SAP97 (10 µg/ml; StressGen). Immunoreactivity was revealed using biotin-conjugated secondary antibodies and the ABC kit (Vector Labs, Burlingame, CA) together with the diaminobenzidine method, and visualized with the aid of a Zeiss microscope (Axioskop) fitted with an LCD camera (DP50-CU; Olympus Co., Japan). All pictures were taken with a 20× objective, at 1/300 s shutter speed using Studio Lite software (Pixera Corp., CA).

### Biotinylation of Surface Proteins and Preparation of Membrane and Cytoplasmic Fractions

Neocortical cultures were treated with 50 ng/ml BDNF for 5 days and then incubated with 1 mM sulfo-NHS-LC-biotin (Pierce) in phosphate-buffered saline containing 1 mM CaCl_2_ and 1 mM MgCl_2_ for 15 min on ice [27]. After washing, cells were lysed and the lysate was incubated with ImmunoPure immobilized streptavidin-beaded agarose (Pierce) overnight at 4°C. Biotinylated proteins were eluted with 2% sodium dodecyl sulfate (SDS) buffer at 95°C and processed for immunoblotting. Membrane and cytoplasmic fractions were prepared as indicated earlier [28].

Crude synaptic/mitochondrial membrane fractions were prepared as previously described [29], [30]. Briefly, cultured neurons (4 sister cultures and 2 independent cultures; n = 8) were collected in lysis buffer (1 ml/10 cm culture dish) and homogenized in 5 ml (final volume) of 0.32 M sucrose solution containing 1 mM EGTA and 0.1 mM leupeptin using a glass-Teflon homogenizer. The homogenate was then centrifuged at 1,000 *g* for 10 min, and the supernatant was collected and centrifuged at 16,000 *g* for 20 min. The pellet was then washed twice by re-suspension in H_2_O containing 1 mM EGTA and 0.1 mM leupeptin, followed by centrifugation at 48,000 *g* for 20 min. Subsequently, the supernatant (cytoplasmic fraction) was used for immunoblotting as indicated below. The pellet (membrane fraction) was re-suspended twice in 50 mM Tris-acetate buffer (pH 7.4) containing 0.1 mM EGTA and centrifuged at 48,000 *g* for 20 min. After the final centrifugation/re-suspension, the membrane fraction was used for immunoblotting. Equal amounts of total membrane and cytoplasmic fractions were resolved by SDS-polyacrylamide gel electrophoresis (SDS-PAGE) and used for immunoblotting.

### GluR1 Knock-down

Four double-stranded RNA oligonucleotides (hereafter referred to as #1 - #4) were used to knock-down GluR1 protein expression in cultured primary neocortical neurons and in HEK293 cells transiently-transfected with pCI mammalian expression vector carrying GluR1 cDNA [31]. They correspond to the following GluR1 cDNA sequences GenBank Accession Number: 1602240A; Reference Sequence: NM_031608.1): 5′-aatcacaggaacatgcggctttt-3′ (#1), 5′-aaaaggagaggctggtggtggtt-3′ (#2), 5′-aaagcctgcggaggcagaggatt-3′ (#3), and 5′-gaagtctgcagaaccatccgtgtt-3′ (#4). GluR1 knock-down was evaluated by immunoblotting and immunostaining and immunostaining data were quantified as indicated in the Supporting Information. Figure S1 shows the quantitation method used to estimate GluR1 protein expression. Following determination of optimal dsRNA concentrations in neurons (Figure S3), DIV 10 primary neuronal cultures were transfected with equal amounts of oligonucleotides #2 and #4 (total dsRNA concentration: 70 nM).

### Construction of Sindbis Viruses and Infection of Cultured Neurons

These viruses were previously described [10], [31]. Sindbis viruses were used to overexpress enhanced green fluorescent protein (EGFP) alone, EGFP fused to carboxyl (C-) terminal sequence of GluR1 (SSGMPLGATGL; single amino-acid letter code) or EGFP fused to the GluR2 C-terminal domain (GYNVYGIESVKI). The infectivity of Sindbis virus particles was titered with BHK cells by serial dilution. Regularly, a viral titer greater than 10^7^ pfu/ml was obtained. DIV 10 cultured neocortical neurons were exposed for 1 h to Sindbis viral vectors, encoding EGFP, EGFP-R1, or EGFP-R2 (titers >10^7^ pfu/ml). After washing, neurons were incubated in 10% FBS-DMEM at 37°C for 24 h in the presence or absence of BDNF. The expression of EGFP was monitored using fluorescence microscopy. At the used viral titers, more than 90% of neurons expressed green fluorescence [10], [31]. BDNF effects on PDZ proteins and AMPA receptors were determined by immunoblotting and reverse transcription and polymerase chain reaction (RT-PCR).

### Deletion Mutagenesis of GluR2

GluR2 C-terminus deletion mutants (Δ5, Δ10 and Δ21 where amino acids 858–862, 853–62, and 842–862 were deleted, respectively) were previously described [27]. Nucleotide deletion was confirmed by DNA sequencing, and the expression of GluR2 deletion mutants was verified by western blotting analysis.

### HEKTrkB Cell Cultures and Plasmid Lipofection

The HEKTrkB cell line was previously described [27]. Briefly, HEK293 cells were stably transfected with pRc/CMV vector carrying mouse TrkB cDNA and were selected in geneticin-containing medium (400 µg/ml geneticin). Drug-resistant colonies were isolated, expanded and maintained in geneticin-containing (200 µg/ml) 10% FBS-DMEM. HEKTrkB cells were plated onto 10 cm culture dishes at a density of 70–80% 24 h prior to transfection. Lipofectamine 2000 (Invitrogen, Carlsbad, CA) or FuGene6 (Roche, Palo Alto, CA) were used according to manufacturers’ recommendations for transient transfection (overnight transfection) with expression vectors carrying the following cDNAs downstream from the CMV promoter: GluR1, GluR2, SAP97, GRIP1, PSD-95 (data not shown) and EGFP [31]. Following transfection, HEKTrkB cells were re-suspended and seeded onto new culture plates. On the next day, they were treated with BDNF (50 ng/mL) for the indicated periods of time. BDNF treatment of HEKTrkB cells increased TrkB phosphorylation.

### Reverse Transcription-polymerase Chain Reaction

Total RNA was extracted from GluR2-, GRIP1-, or EGFP-transfected HEKTrkB cells that were treated, or not, with BDNF (50 ng/mL) for 1, 2 or 3 days was done as previously described [31]. Using an RT-PCR High kit (Toyobo), cDNA fragments were amplified within the linear amplification range. The following primers were used: 5′-ACCATGTGAAAATTCAGAGG-3′ and 5′-ATTCCAAAGCCAGTGACAGG-3′ generating a 415-bp fragment for GRIP1 (20 cycles); 5′-AGCCCTCTGTGTTTGTGAGGA-3′, 5′-CAATAGTGGCTGCGGAAACGGG-3′ generating a 192-bp product for GluR2; 5′-CACAGCTGAGAGGGAAATCG-3′ and 5′-CACACAGAGTACTTGCGCTC-3′ generating a 348-bp product for endogenous β-Actin. The PCR products were electrophoresed through a 2% agarose gel, stained with SyberGreen I (Molecular Probes), and imaged with a CCD camera (Cosmicar; Pentax).

### Co-immunoprecipitation and Immunoblotting

Cultured neurons or HEKTrkB cells were lysed using sample buffer [10% glycerol, 2% sodium dodecyl sulfate (SDS), 10 mM Tris-HCl, 140 mM Tris–HCl, pH 7.5] or immunoprecipitation buffer (10 mM Tris–HCl, 65 mM NaCl, 1 mM EDTA, 0.5 EGTA and 1% deoxycholate, pH 7.5) supplemented with protease and phosphatase inhibitors [31]. Typically, 3–10 µg of anti-N-terminal GluR1 [24], anti-N-terminal GluR2 (Millipore), anti-SAP97 [31], anti-GRIP1 (Millipore) or anti-Pick1 [10] antibodies, which had been pre-adsorbed to Protein A/Protein G–sepharose beads, were incubated overnight with 200–400 µg of total cell lysate at 4°C and washed extensively. Immunoprecipitated, total cellular protein, or protein from membrane and cytoplasmic fractions were denatured by boiling in sample buffer containing 100 mM dithiothreitol and 5–10% 2-mercaptoethanol for 5–10 min, separated by SDS-PAGE and blotted onto PVDF membranes (Bio-Rad, Hercules, CA). Primary antibodies (see below) were then incubated with membranes at 4°C overnight. Immunoreactivity was detected with goat anti-rabbit (DAKO, Kyoto, Japan) or goat anti-mouse (Jackson Laboratories, Bar Harbor, ME) antibodies conjugated to peroxidase (diluted 1∶10000) followed by chemiluminescence reaction (ECL kit; GE Healthcare) and film exposure. For immunoblotting, all of the following primary antibodies were used at 1 µg/ml except for Pick1 which was used at 2 µg/ml: anti-SAP97 monoclonal (StressGen) [31], anti-GRIP1 (Millipore), anti-Pick1 [10], and anti-GluR2/3 (Millipore). Anti-Actin (Boehringer Mannheim and Millipore), p-TrkB, and TrkB antibodies (Cell Signaling), were all used at 1∶1000 dilution. Specificity of the immunoreactivity was determined by comparing detected band sizes with the reported molecular weights. Biotinylated proteins were eluted with 2% SDS buffer at 95°C and processed for immunoblotting.

### Statistical Analysis

Student’s *t*-test was used to analyze differences in immunoreactivity when the number of samples per set included more than three. Results were expressed as means ± standard deviation (SD), and the number of similar experiments is indicated in each figure legend.

## Results

### Prolonged BDNF Treatment Upregulates Total and Membrane-bound Levels of AMPAr Subunits in Cultured Primary Cortical Neurons

**Figure 1:**
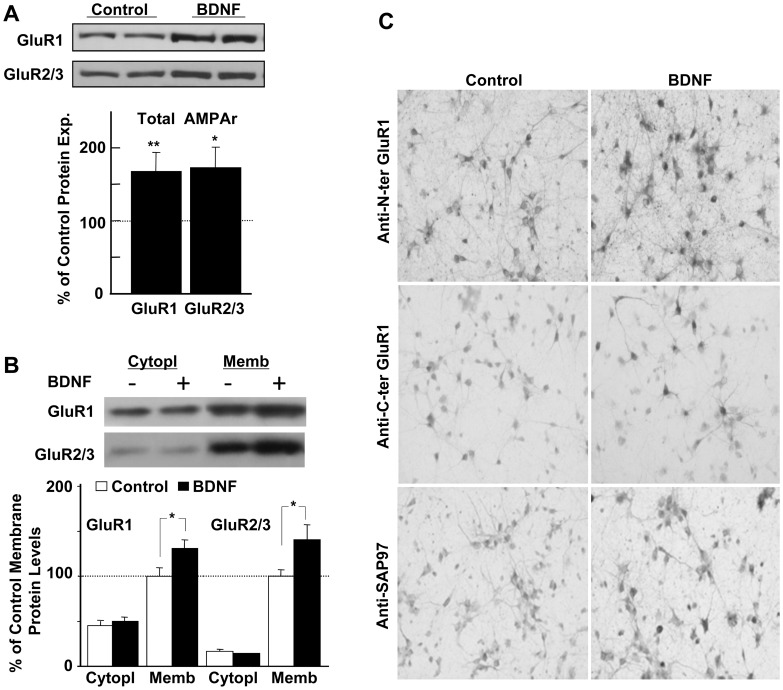
Prolonged BDNF treatment of cultured neocortical neurons enhances GluR1- and SAP97-like immunoreactivities and increased AMPAr subunit expression on neuronal cell membrane. Cultured neurons are treated with BDNF (50 ng/ml), or not (control) for 4–5 days. At the end of treatment, neurons are lysed and probed with anti-GluR1 and anti-GluR2 using total cell lysates (A) and cytoplasmic and membrane fractions (B). Results were obtained from 8 experiments (A, B) *: *p*<0.05; **: *p*<0.005. C) Similarly treated cultures, (BDNF (50 ng/ml), or not (control) for 4–5 days, were stained with the indicated antibodies: Upper panels, anti-N-terminal domain of GluR1; middle panels, anti-C-terminal domain of GluR1; lower panels, anti-SAP97.

Neuronal cultures were treated with BDNF (50 ng/mL) or vehicle (equal volume of 2 mg/mL bovine serum albumin in phosphate buffered saline) daily for 5 days. At the end of treatment, cells were lysed and processed for immunoblotting. BDNF treatment increased total protein levels of AMPAr subunits, GluR1 and GluR2/3 compared to untreated sister cultures (Fig. 1A). Similar cultures were collected and differentially fractionated to isolate the membrane and cytoplasmic fractions [29], [30]. Protein samples were then processed for immunoblotting with equal total protein amounts loaded in all gel lanes (Fig. 1B). The results showed BDNF-mediated upregulation of AMPAr subunits in the membrane fraction but not in the cytoplasmic fraction, indicating that BDNF-dependent upregulation of these subunits is associated with their localization in the membrane fraction.

These results were further confirmed using immunostaining (Fig. 1C). Primary neuronal cultures were treated daily with BDNF (50 ng/ml) for 4–5 days, fixed at DIV 11–12, permeabilized and labeled with anti-N-terminal GluR1 [24], anti-C-terminal GluR1 antibodies (Millipore), or with anti-SAP97 [21], [31]–[34]. The results indicated that BDNF treatment increases GluR1-like and SAP97-like staining (Fig. 1C); compared to control cultures, SAP97-like immunoreactivity also had a BDNF-dependent intensification showing many SAP97-positive neurites. The BDNF-treated cultures displayed enhanced GluR1-like immunoreactivity irrespective of the used GluR1 antibody despite showing markedly different staining patterns with the two anti-GluR1 antibodies: The anti-N-terminus GluR1 antibody stained neurons more efficiently than the anti-C-terminus GluR1 antibody; GluR1 staining was better demonstrated with the anti-N-terminal antibody covering the soma and dendritic processes, indicative of high GluR1 expression in these subcellular compartments. Conversely, strong labeling with the anti-C-terminal antibody was rather restricted to the soma, with poor staining in distal dendrites [35]. Similar results were obtained for labeling primary neuronal cultures with GluR2 and GRIP1 antibodies (data not shown).

### Acute BDNF Treatment Increases GluR1-SAP97 and GluR2-GRIP1 Interactions in Neurons

**Figure 2:**
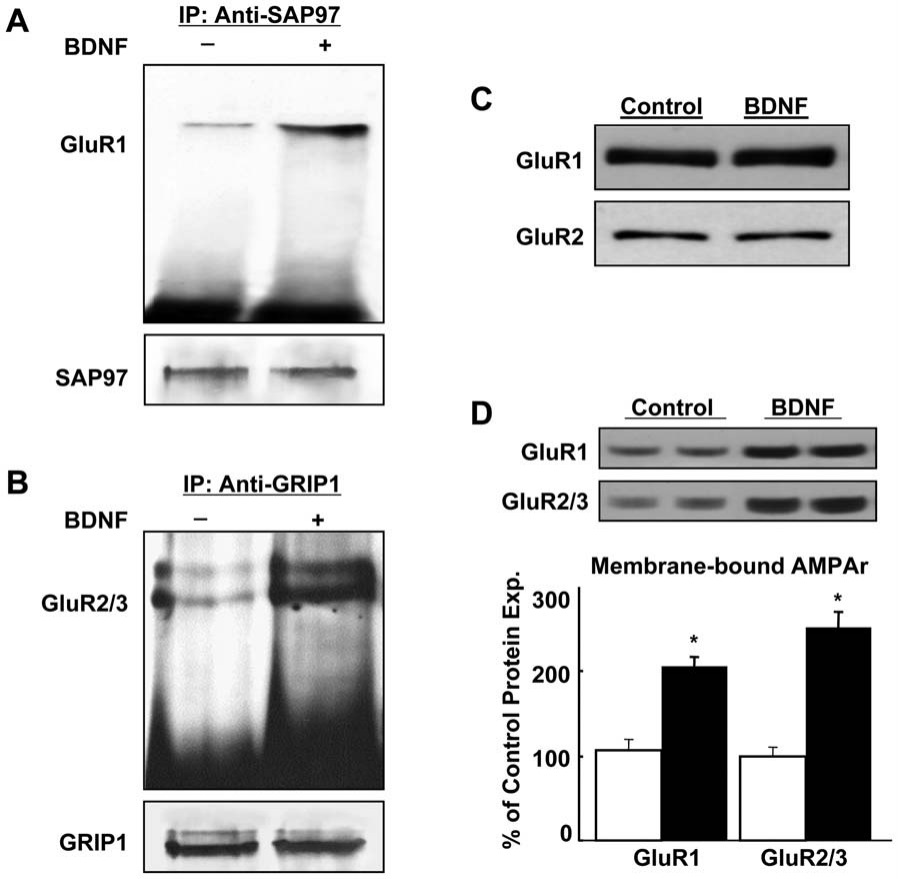
Acute BDNF treatment increases GluR1-SAP97 and GluR2-GRIP1 interactions in neurons and enhances GluR1 and GluR2 expression on neuronal cell surface. DIV 14 cultured neurons acutely-treated with BDNF (1 h, 50 ng/ml), or not, are lysed and processed for co-immunoprecipitation using anti-SAP97 (A) and anti-GRIP1 (B). Immunoblots are probed with anti-GluR1 and anti-GluR2 (upper blots in A and B, respectively). Re-probing the same blots with anti-SAP97 and anti-GRIP1 indicates equal loading (lower blots in A and B, respectively). C) Acute BDNF treatment (1 h, 50 ng/ml) does not increase total GluR1 and GluR2 protein levels in similarly treated cultures. D) Similarly treated cultures, as in (C), are treated with biotinylation reagent and biotinylated total surface proteins are analyzed with anti-GluR1 and anti-GluR2 antibodies. Results are representative of 4 experiments; *: *p*<0.01; Student’s *t*-test.

Our previous results indicated that chronic exposure of primary neuronal cultures to BDNF increased GluR1-SAP97 and GluR2-GRIP1 interactions [31]. We then asked whether acute BDNF (1 h) treatment of cultured cortical neurons could rapidly upregulate the interactions between GluR1 and GluR2 with SAP97 and GRIP1, respectively. DIV14 neurons were thus exposed to BDNF (50 ng/ml) for 1 h. Samples were then collected and processed for co-immunoprecipitation. The results (Fig. 2A, B) illustrated rapid upregulation of GluR1-SAP97 and GluR2-GRIP1 interactions. These interactions were therefore distinct from the ones we had previously shown in that they were not accompanied by BDNF-mediated upregulation of AMPAr subunits and of their scaffolding proteins (Fig. 2C) [31]. In addition, DIV10-14 primary neuronal cultures were treated with BDNF (1 h, 50 ng/ml) or vehicle and subjected to biotinylation in order to quantify the total amount of AMPAr subunits found at neuronal cell surface. The results clearly indicated that acute BDNF treatment increased biotinylated, surface-bound GluR1 and GluR2 (Fig. 2D). Together, these results indicated that acute BDNF treatment specifically increased the interaction of each AMPAr subunit with its scaffolding protein and the levels of AMPAr subunits at the neuronal cell membrane [27].

### GluR1 Knock-down in Cultured Cortical Neurons

**Figure 3:**
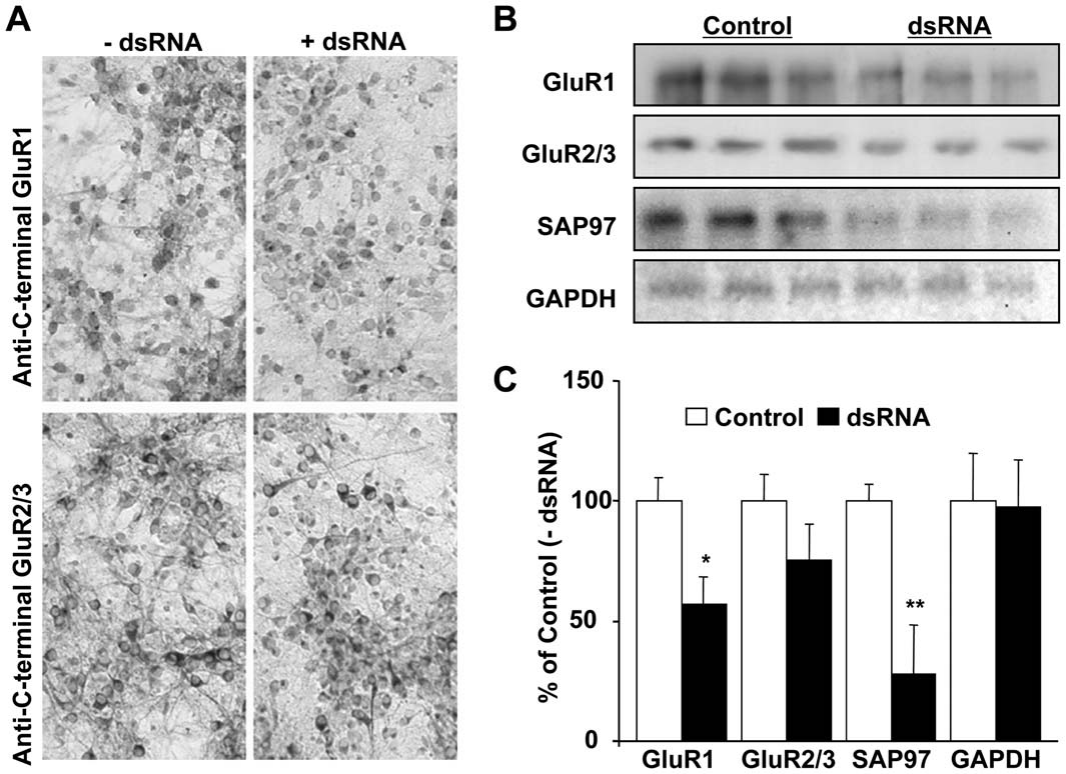
GluR1 knock-down in cultured cortical neurons. A) GluR1-specific dsRNA reduces GluR1-like but not GluR2-like immunostaining in transduced neurons. B) Immunoblots show specific decline of GluR1 immunoreactivity in cultured neurons transduced with GluR1-specifc dsRNA; GluR2/3 levels are slightly reduced but reduction is not statistically significant. SAP97 protein levels show a highly significant decline following GluR1-specific dsRNA transduction. C) Quantification of 6 experiments carried out as in B. *p<0.05, **p<0.01; Student’s *t*-test.

Next, we sought to determine the consequences of reducing GluR1 protein levels on its partner proteins. Immunocytochemistry and immunoblotting were used to quantify GluR1 and GluR2 immunoreactivities in dsRNA- and mock-transfected neurons (Figures S1, S2, S3, and Fig. 3). Preliminary experiments were conducted in GluR1-transfected HEK293 cells to determine the efficacy of 4 distinct dsRNA oligonucleotides, used individually or in combination, to knock-down GluR1 (Supporting Information). An example of the method used to quantify the knock-down efficacy of GluR1-specific dsRNA oligonucleotides is provided (Figure S1). Based on results indicating that oligonucleotides #2 and #4 were the most efficient when used in combination in reducing exogenous GluR1 protein levels in HEK293 cells (Figure S2), we thus decided to use them in the remaining experiments in neurons (Fig. 3). Additional experiments were conducted in cultured neurons to determine the optimal concentrations of dsRNA oligonucleotides and maximal concentrations of transfection reagents tolerated by neurons for each individual experiment (Figure S3). A concentration of 70 nM oligonucleotides #2 and #4 yielded reproducible and a statistically significant reduction of GluR1.

Equal amounts of oligonucleotides ^#^2 and ^#^4 were used together to knock-down GluR1 protein in neurons (Fig. 3). Immunostaining of cultured neurons with anti-C-terminal and anti-N-terminal GluR1 antibodies demonstrated reduced GluR1-like staining in dsRNA-transfected neurons, as compared to mock-transfected cells. Immunoblotting results indicated significant reduction in total GluR1 protein expression (52±18%; *p*<0.05), but only a slight and insignificant reduction of GluR2/3 expression as in GluR1-specific dsRNA-transfected neurons compared to mock-transfected cells. Remarkably, we also observed a highly significant decline in SAP97 protein levels in neurons transfected with GluR1-specific oligonucleotides (Fig. 3B–C). GluR1, GluR2/3, SAP97 and GRIP1 protein levels were not altered in neurons transfected with GAPDH-specific dsRNA (data not shown).

### Overexpression of GluR1 and GluR2 C-terminal Decoys Abrogates BDNF-dependent Upregulation of AMPAr Subunits and AMPAr-scaffolding Proteins

Our previous results implicated PDZ proteins in BDNF-dependent upregulation of GluR1 and GluR2: Sindbis virus-mediated overexpression of chimeric protein decoys made of the C-terminal domains of GluR1 and GluR2 fused to enhanced green fluorescent protein (EGFP-R1 and EGFP-R2, respectively) specifically abrogated BDNF-dependent upregulation of the corresponding AMPAr subunits. In addition, co-immunoprecipitation results indicated that the same decoys eliminated the BDNF-mediated enhanced interactions of GluR1-SAP97 and GluR2-GRIP1 [31].

**Figure 4:**
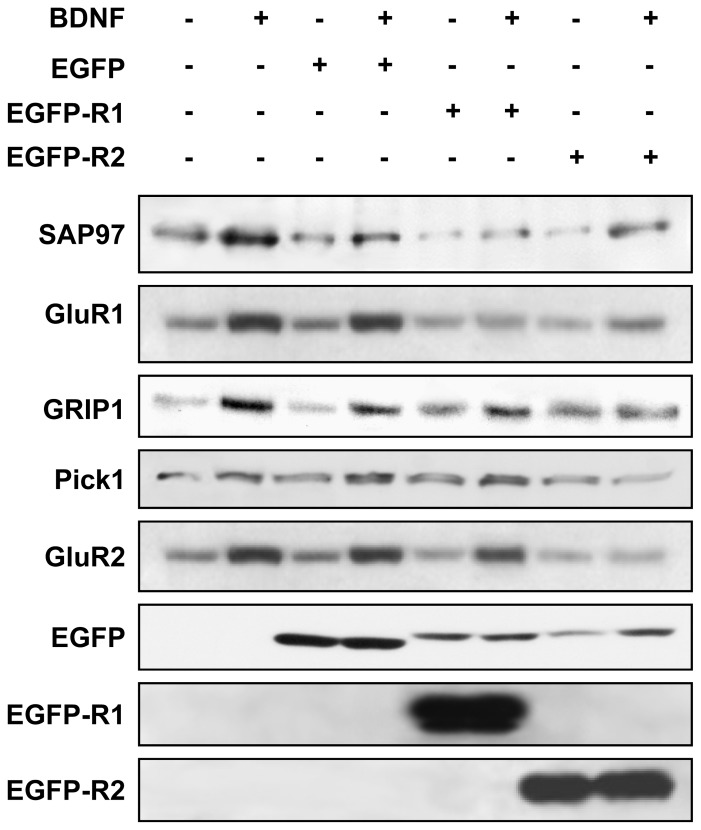
Over-expression of GluR1 and GluR2 C-terminal decoy peptides eliminates BDNF-mediated upregulation of AMPAr subunits and their scaffolding proteins. DIV 10 cultured cortical neurons are infected with a control Sindbis virus to express EGFP alone or with viruses encoding EGFP-R1 and EGFP-R2 and treated with BDNF for 1 day or not. Protein samples are extracted and analyzed by immunoblotting as indicated in “Materials and Methods”. Over-expression of the decoy peptides, corresponding to the C-terminal domains of GluR1 and GluR2, results in specific elimination of the up-regulatory effects of BDNF on the corresponding AMPAr subunit and its interacting partner PDZ protein(s).

Considering the highly significant and rapid down-regulation of SAP97 observed in neurons transfected with GluR1-specific dsRNA oligonucleotides (Fig. 3), we next examined whether over-expression of the chimeric protein EGFP-R1 would compete with native GluR1 for binding to SAP97 and abolish not only BDNF-mediated up-regulation of GluR1, but also BDNF-dependent upregulation of SAP97 protein levels. The results revealed that overexpression of EGFP-R1 did indeed eliminate BDNF-mediated upregulation of both SAP97 and GluR1 proteins (Fig. 4). Similarly, we evaluated the effects of overexpressing EGFP-R2 on BDNF-mediated increase of GluR2-interacting PDZ proteins GRIP1 and Pick1. Again, overexpression of EGFP-R2 abolished BDNF-induced upregulation of GluR2/3, GRIP1, and Pick1 (Fig. 4). Importantly, overexpression of EGFP and EGFP-R2 did not affect BDNF-dependent upregulation of SAP97 protein expression in a non-specific way, as the BDNF effects on SAP97 were still present in both EGFP and EGFP-R2 overexpression conditions. Likewise, EGFP-R1 overexpression did not alter BDNF-dependent upregulation of GluR2/3, GRIP1 and Pick1. Therefore, overexpression of AMPAr subunit-specific decoys eliminated BDNF-dependent upregulation of the PDZ proteins that specifically interact with the corresponding AMPAr subunit, implying that GluR1 and GluR2 interactions with their scaffolding PDZ proteins are essential to increase expression of both AMPAr subunits and scaffolding proteins.

### Chronic BDNF Treatment of HEKTrkB Cells does not Upregulate the Expression of Heterologous Proteins

**Figure 5:**
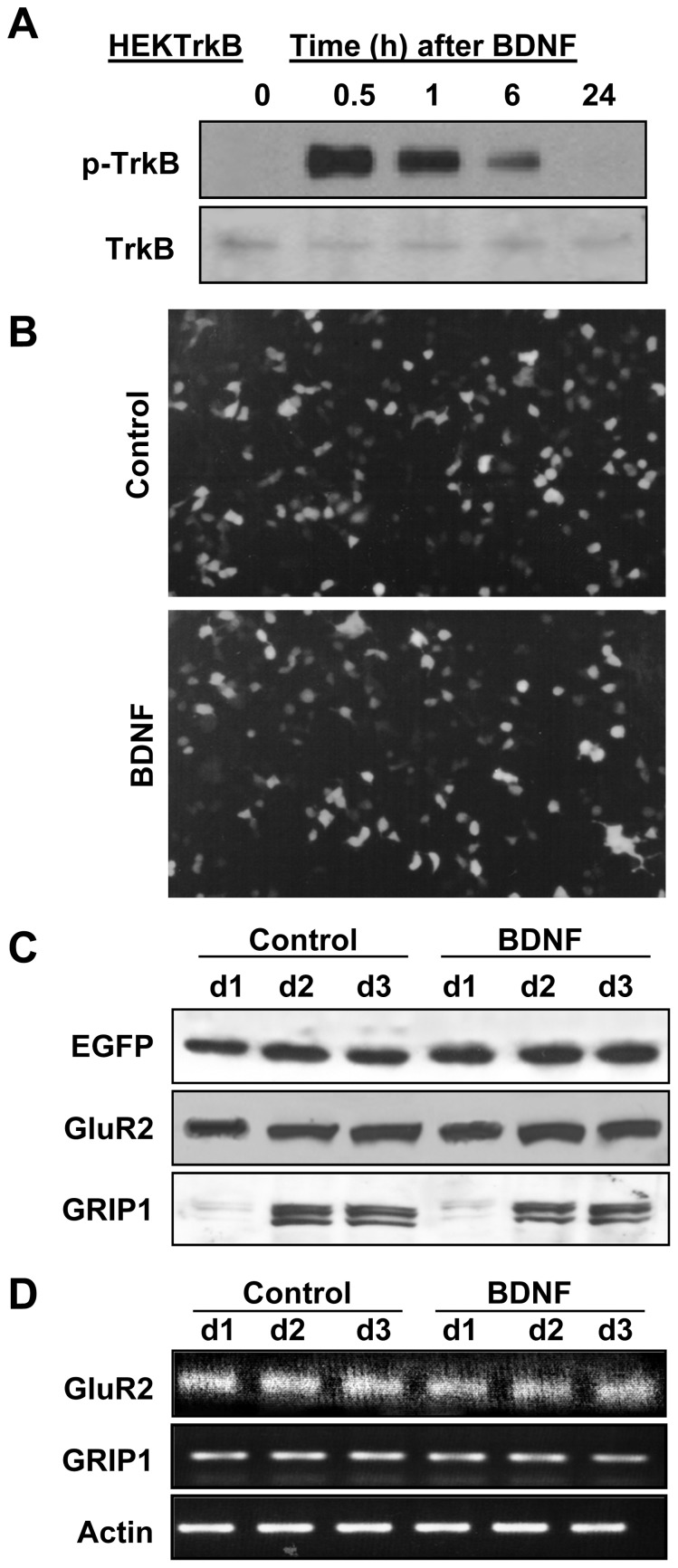
Effects of prolonged BDNF treatment on heterologous proteins expressed in HEKTrkB cells. A) BDNF treatment leads to TrkB phosphorylation (p-TrkB) in HEKTrkB cells. Cells are treated with BDNF (50 ng/ml) for 1 h after which culture medium is replaced with fresh medium. Cells are incubated for the indicated times before being lysed and used for analysis with TrkB and p-TrkB antibodies. B) HEKTrkB cells are transfected overnight with pCMV-EGFP (31). Cells were re-suspended and split into two equal numbers before being re-plated and treated with BDNF (50 ng/mL, daily for 3 days). Similar levels of EGFP fluorescence is indicative of similar expression levels in BDNF-treated or untreated HEKTrkB cells. C) Cells are treated as in (B); protein samples are collected from cells treated with BDNF for 1, 2, or 3 days or not and analyzed by immunoblotting for EGFP, GluR2 and GRIP1 protein contents. The results show that chronic BDNF treatment in HEKTrkB cells does not increase the expression of any of the heterologous proteins. D) Cells are transfected as in (C); total RNA is extracted from cells treated with BDNF or vehicle (control) for 1, 2, or 3 days and analyzed by RT-PCR. The results indicate that similar levels of exogenously-expressed mRNA (GluR2 and GRIP1) are present in BDNF-treated and untreated HEKTrkB cells. Amplification of endogenously-expressed Actin mRNA serves as an internal control.

We previously showed that SAP97 and GluR1 coexpression in regular HEK293 cells was sufficient to increase the total expression of both proteins beyond their levels when individually expressed. However, co-expression of GRIP1 with GluR2 in the same cell line did not increase either one of them [31]. In the next experiment, we sought to understand the mechanisms underlying this discrepancy and hypothesized that BDNF-TrkB signaling might be required. To address this issue, we used a heterologous expression system comprised of HEK293 cells that express stable levels of the BDNF receptor TrkB (HEKTrkB) to determine if acute BDNF treatment of cells co-transfected with GluR2 and GRIP1 could enhance their interaction and lead to their increased accumulation. We first confirmed that brief BDNF treatment of these cells could elicit TrkB phosphorylation; the results indicated that exposure of these cells to BDNF (1 h; 50 ng/mL) did increase TrkB phosphorylation that lasted beyond the removal of BDNF with maximal TrkB phosphorylation detected at 30 min after BDNF removal and gradually declining to untreated control levels by 18–24 h (Fig. 5A). Next, we examined the effects of prolonged BDNF treatment on the expression of heterologous individually-expressed proteins in HEKTrkB cells and evaluated the levels of exogenous EGFP, GRIP1 and GluR2 expressed from mammalian expression vectors with a CMV promoter [31]. EGFP levels were also monitored using fluorescence microscopy following transfection into HEKTrkB cells and treatment with BDNF (50 ng/mL; once daily for 2 days); 48 h post-transfection the fluorescence intensity and number of EGFP-expressing HEKTrkB cells were equivalent in BDNF-treated and untreated conditions (Fig. 5B). Transfected HEKTrkB cells were treated with BDNF (50 ng/mL) for 1, 2 and 3 days. Expectedly, this treatment did not upregulate the expression of the heterologous, transiently-expressed EGFP, GRIP1 and GluR2, as their protein levels were equivalent in BDNF-treated and untreated HEKTrkB cells (Fig. 5C). These results were further confirmed using reverse transcription and polymerase chain reaction (RT-PCR). mRNA was extracted from HEKTrkB cells that were transfected with GluR2 and GRIP1 vectors and treated with BDNF or vehicle for 1, 2 or 3 days, exactly as for Fig. 5C; RT-PCR data (Fig. 5D) showed equivalent levels of amplified GluR2 and GRIP1 PCR products from BDNF- or vehicle-treated HEKTrkB cells (Fig. 5D). RT-PCR products of endogenous β-actin (control) were also equivalent in all of these conditions. Thus, compared to untreated controls, BDNF did not affect EGFP, GluR2 and GRIP1 protein levels throughout the 3 days of treatment. Therefore, in contrast to results from BDNF-treated cultured cortical neurons these results indicated that prolonged BDNF treatment in this heterologous system could not enhance the expression levels of individually-expressed GluR2 and GRIP1 [36].

### Acute BDNF Treatment Increases Interaction and Protein Levels of Coexpressed GluR2 and GRIP1 in HEKTrkB Cells

**Figure 6:**
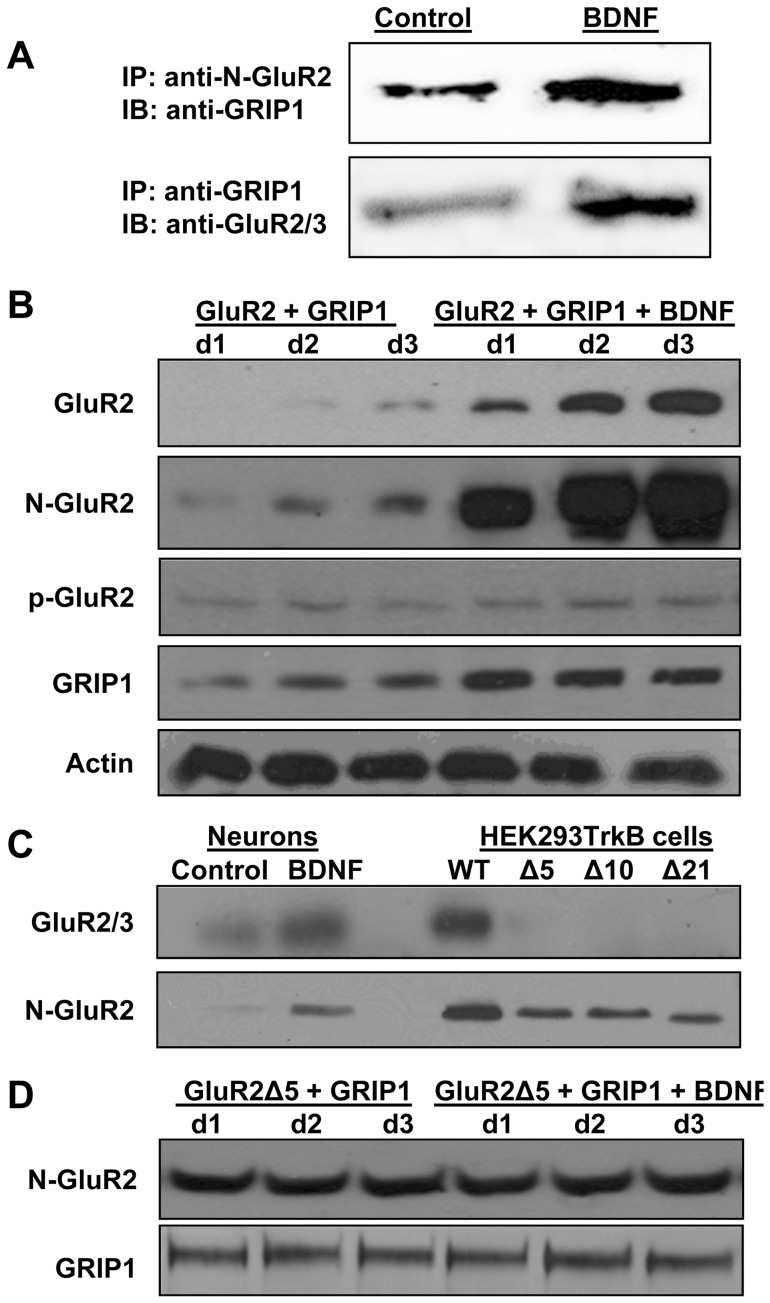
BDNF treatment of HEKTrkB cells increases GluR2-GRIP1 interaction and upregulates their total protein levels. A) Upper panel, immunoprecipitation is done with anti-N-terminal GluR2 antibody and immunoblotting with anti-GRIP1; lower panel, immunoprecipitation is done with anti-GRIP1 and immunoblotting with anti-C-terminal GluR2/3 antibody. The results indicate increased interaction between GluR2 and GRIP1 following 1 h BDNF treatment (50 ng/mL) of HEKTrkB cells cotransfected with GluR2 and GRIP1 expression vectors. B) HEKTrkB cells are transfected with WT GluR2 and GRIP1. One day after transfection, cells are treated with BDNF or not for 1, 2, or 3 days. Protein samples are analyzed for the total protein content of GluR2 and GRIP1 as well as for phospho-GluR2 (p-GluR2-Ser880). C) Western blotting of proteins samples from cultured neurons treated with BDNF or not were run in parallel with samples extracted from HEKTrkB cells that were transfected with wild type (WT) or deletion mutant of GluR2 lacking the last 5, 10, or 21 aminoacids (Δ5, Δ10, and Δ21, respectively). The C-terminal antibody against GluR2/3 only labeled GluR2 WT. The N-terminal anti-GluR2 labeled all constructs. D) HEKTrkB cells were transfected with GluR2Δ5 together with GRIP1. The results indicate gradual accumulation of WT GluR2 but not GluR2Δ5 in samples treated with BDNF.

Next, we determined if brief BDNF treatment (1 h; 50 ng/mL) of HEKTrkB cells could increase the interaction of transiently co-transfected GluR2 and GRIP1. Co-immunoprecipitation results showed that 1 h BDNF treatment significantly increased GluR2-GRIP1 interaction in HEKTrkB cells ∼6 fold as compared to untreated cells (Fig. 6A). Thus, the use of this heterologous expression system further confirmed the acute effect of BDNF on GluR2-GRIP1 interaction that was observed in neurons (Fig. 2).

Previously, we found that co-expression of GluR2 with GRIP1 in regular HEK293 cells did not result in their mutual stabilization (31). Taking into consideration the results of the previous experiments (Fig. 5, 6A), we next asked whether BDNF-dependent upregulation of GluR2 interaction with GRIP1 could lead to increased GluR2 and GRIP1 protein levels (accumulation) in BDNF-treated HEKTrkB cell. To address this issue, HEKTrkB cells were co-transfected with GluR2 and GRIP1 expression plasmids for 24 h. Cells were then re-suspended, split equally, and re-plated onto 6 plates. Three plates received BDNF treatment (50 ng/mL) for 1, 2, or 3 days and the remaining 3 plates were treated with vehicle for similar durations. Results in Fig. 6B show immunoblotting data with anti-GluR2 C-terminal and anti-GluR2 N-terminal antibodies of samples extracted from HEKTrkB cells that were treated with BDNF (50 ng/mL) or vehicle for 1, 2, and 3 days. The results indicated upregulation of GluR2 protein levels with prolonged BDNF treatment. Results for GRIP1 also showed BDNF-dependent accumulation, as compared to the vehicle-treated conditions. Probing the same samples with anti-phospho-GluR2 antibody, which recognizes the phosphorylated serine 880 epitope of GluR2, indicated that BDNF treatment did not increase GluR2 phosphorylation. β-actin antibody was used to confirm equal protein loading and the data indicated equivalent amounts of protein in all lanes. Taken together, these results indicated accumulation of co-expressed GluR2 and GRIP1 proteins following prolonged BDNF treatment.

Results were also obtained from HEKTrkB cells that were co-transfected with GRIP1 together with GluR2 C-terminal deletion mutants GluR2Δ5, GluR2Δ10 and GluR2Δ21 lacking the last 5, 10 or 21 amino acids [27]. N-terminal and C-terminal GluR2 antibodies were used to confirm the expression of these deletion mutants in HEKTrkB cells in parallel to samples extracted from BDNF- or vehicle-treated neuronal cultures (Fig. 6C). Compared to vehicle-treated controls, BDNF treatment of neuronal cultures (50 ng/mL/day; 4 day treatment) resulted in increased labeling with both antibodies in BDNF-treated samples. Expectedly, the anti-C-terminal GluR2 antibody (a.k.a. GluR2/3 antibody) could only recognize the full length GluR2 because the recognized epitope was either partially or completely missing from the deletion constructs. The anti-N-terminal GluR2 antibody labeled proteins extracted from HEKTrkB cells that were transfected with full length GluR2, GluR2Δ5, GluR2Δ10 and GluR2Δ21 with the GluR2Δ21 signal showing slightly smaller molecular weight (Fig. 6C). The GluR2Δ5 mutant lacks the epitope required for interaction with partner PDZ proteins, including GRIP1 and Pick1 [10], [27], [37], [38]. Therefore, we used the GluR2Δ5 deletion mutant in the next experiment to further elucidate the role of GluR2-GRIP1 interaction in mediating BDNF-dependent accumulation of GluR2 and GRIP1 proteins. HEKTrkB cells were co-transfected with GRIP1 and GluR2Δ5 and treated with BDNF or vehicle for 1, 2, or 3 days, as indicated earlier. Protein samples were collected and analyzed for total amounts of GluR2Δ5 and GRIP1 expressed in these cells. The results (Fig. 6D) revealed that BDNF treatment did not lead to accumulation of GluR2Δ5. In parallel, GRIP1 protein levels were not altered in BDNF-treated samples, as compared to vehicle controls, showing constant protein levels from day 1 to day 3. Therefore, BDNF treatment of HEKTrkB cells caused upregulation of full length GluR2 but not GluR2Δ5. In addition, GRIP1 protein levels were upregulated (stabilized) only in the presence of full length GluR2. Taken together with the results in Figure 5, these findings indicated that GluR2-GRIP1 interaction requires BDNF signaling and that the resulting interaction mediated BDNF-dependent upregulation of both proteins. Notably, these results were in line with the elimination of BDNF-mediated upregulation of GRIP1 seen in cultured neurons with overexpression of the EGFP-R2 decoy (Fig. 5).

## Discussion

Neurotrophins and cytokines play fundamental roles in glutamatergic synapse development and plasticity. BDNF treatment upregulates the expression of AMPAr subunits and their associated PDZ proteins (AMPAr compartment) and enables synaptic plasticity associated with AMPAr subunits and their scaffolding proteins in various types of neurons but has little or no effects on NMDA receptors [25]–[27], [31], [32], [39]–[42]. Other trophic factors and cytokines including bFGF, EGF, and TGFα exert effects opposite to those of BDNF on AMPAr subunits and their associated scaffolding proteins [34], [43], [44]. AMPAr subunit stabilization at synapses is critical for the maintenance of long term encoding of synaptic events, such as those involved in late phase long term potentiation (l-LTP) and persistence of memory [14], [15], [45]–[47]. Various studies demonstrated important functions for PDZ proteins, including stabilization of AMPAr subunits to mediate synaptic changes associated with long term modulation of synaptic activity; this has previously been referred to as synaptic consolidation [1], [2], [3], [11], [47]. Although acute BDNF treatment of neurons increases surface expression of AMPAr complexes in a GluR2-dependent way, it remains unclear how BDNF contributes to long term stability and accumulation of synaptic proteins and receptors involved in synaptic development, and lasting potentiation [27], [42], [47], [48].

We have previously shown that coexpression of GluR1 and SAP97 in regular HEK293 cells upregulates both proteins beyond their levels when individually expressed and that prolonged BDNF treatment of cultured neurons increases their interactions, implying that GluR1 and SAP97 coexpression leads to their mutual stabilization [31]. However, the enhanced GluR1-SAP97 interaction in neurons may be an artifact caused by the concomitant upregulation of SAP97 and GluR1 protein levels with prolonged BDNF treatment [31]. So as to avoid possible confounding effects of BDNF on the expression levels of these proteins, we used acute (1 h) BDNF treatment and showed that this treatment specifically increases GluR1-SAP97 interaction without increasing GluR1 and SAP97 protein levels (Fig. 3). Notably, the immunostaining results (Fig. 1C) agree with the co-immunoprecipitation data; the GluR1 C-terminal epitope, recognized with the corresponding antibody, is the same motif which interacts with SAP97 and this epitope appears to be masked because of its binding to SAP97, at least in distal dendrites. A previous report indicates that GluR1-SAP97 interaction occurs early in the secretory pathway, starting at the level of the endoplasmic reticulum and the *cis*-Golgi compartments [35]. The discrepancy in GluR1 staining patterns following prolonged BDNF treatment with the two anti-GluR1 antibodies also infers that BDNF treatment in neurons is associated with enhanced GluR1-SAP97 interaction. Moreover, the GluR1 knock-down experiment (Fig. 4) indicates that GluR1-SAP97 interaction is essential for the accumulation/stabilization of SAP97. Interestingly, GluR1 knock-down does not significantly reduce GluR2 protein levels despite its known interaction with GluR1 [49]. Results from the EGFP-R1 decoy experiment provide further support for the mutual stabilization of GluR1 and SAP97 as BDNF-mediated upregulation of both GluR1 and SAP97 is abrogated with EGFP-R1 overexpression (Fig. 5). Although the current results do not address the question of BDNF-mediated increase in GluR1 and SAP97 in neurons, as BDNF increases GluR1 and SAP97 protein levels without increasing their mRNA levels, it is still possible that BDNF treatment could increase their mRNA translation [25]–[27], [31], [50], [51]. Indeed, rapid GluR1 protein upregulation as a consequence of increased mRNA translation has recently been reported following BDNF treatment and after acute antidepressant treatment with ketamine [52]–[54]. Protein translation of PSD-95, another SAP97-like PDZ protein that also interacts with GluR1, is also enhanced following ketamine treatment [52], [54]. Accordingly, the previous and current results imply that BDNF-mediated upregulation of GluR1 and SAP97 proteins in neurons requires their interaction, but that their upregulation is secondary to their enhanced expression in an as yet undetermined BDNF-dependent way. Taken together, our results indicate that stabilization of GluR1 and SAP97 is indeed mutual and bidirectional because GluR1 knock-down also results in SAP97 down-regulation and because interference with their interaction eliminates their BDNF-dependent upregulation. The current results are significant because GluR1-SAP97 interaction can be implicated in GluR1 intracellular trafficking and in GluR1 protein stabilization [2], [52], [55]. The present findings also agree with previous studies showing tight co-regulation and close dependence between GluR1 and SAP97 at the protein level [31], [32]. These new results can be relevant for schizophrenia, Parkinson’s disease and Alzheimer’s disease, as they provide a correlation between diminished BDNF-TrkB signaling and the observed declines in GluR1 and SAP97 proteins in these diseases [18]–[20], [56]–[58]. Future studies should examine BDNF effects on GluR1 and SAP97 translation and degradation.

Previously, we have shown that, in contrast to coexpression of GluR1 and SAP97, heterologous coexpression of GluR2 and GRIP1 in regular HEK293 cells failed to increase the levels of both proteins beyond their levels when individually expressed. Thus, GluR1 and SAP97 mutually enhanced the protein levels of one another, while GluR2 and GRIP1 did not, at least in HEK293 cells [31]. It is therefore evident that a divergence exists in mechanisms regulating GluR1-SAP97 and GluR2-GRIP1 interactions and stabilization. We have also shown that prolonged BDNF treatment of cultured neurons increased GluR2-GRIP1 interaction; yet this was associated with confounding increased expression of both proteins in neurons [31]. The current results indicate that acute (1 h) BDNF treatment also increases GluR2-GRIP1 interaction but without the confounding effect of BDNF-mediated upregulation of the levels of both proteins. In the current study, we thus examine the role of both acute and prolonged BDNF treatments in promoting GluR2-GRIP1 interaction and increasing their total protein levels and we hypothesize that upregulation of GluR2-GRIP1 interaction and protein level requires BDNF-TrkB signaling. To examine this hypothesis, we use a heterologous expression system consisting of HEKTrkB cells transfected with expression vectors carrying the cDNAs of GluR2 and GRIP1 to isolate the BDNF effects on GluR2-GRIP1 interaction and protein accumulation/stabilization from confounding transcriptional and translational effects that can be induced by BDNF-TrkB signaling in cultured neurons. As can be seen in the results (Fig. 5, 6), prolonged treatment (up to 3 days) with BDNF does not increase mRNA and protein levels of GluR2 and GRIP1 when expressed individually in HEKTrkB cells, but acute exposure to BDNF increases GluR2-GRIP1 interactions (Fig. 6A), replicating the acute effects of BDNF in neurons (Fig. 3). Furthermore, this BDNF-driven GluR2-GRIP1 interaction is associated with increased GluR2 and GRIP1 protein levels, implying BDNF-mediated stabilization of both proteins (Fig. 6B). In agreement, GluR2Δ5 coexpression with GRIP1 fails to cause upregulation of GRIP1 or GluR2Δ5 in response to BDNF treatment (Fig. 6D), indicating that GluR2- GRIP1 interaction is required to mediate BDNF-driven upregulation/stabilization of both proteins. Notably, overexpression of EGFP-R2 to compete with native GluR2 eliminates GluR2-GRIP1 interaction and abrogates BDNF-mediated upregulation of both GluR2 and GRIP1 (Fig. 5) [31]. The present results and our previous findings can be illustrated schematically to show the observed differences in the regulation of GluR1-SAP97 and GluR2-GRIP1 (Figure S4).

GRIP1, GRIP2/ABP, and Pick1 interact with the C-terminal domain of GluR2/3. The phosphorylation of GluR2 Ser880 by protein kinase C plays a crucial role in determining the GluR2 binding partner; GluR2 Ser880 phosphorylation favors binding to Pick1 and hinders interaction with GRIP1 or GRIP2/ABP. In addition, GluR2-Pick1 interaction has been implicated in AMPAr endocytosis and correlated with the induction of synaptic long term depression (LTD) [10], [59]. In opposition, BDNF treatment upregulates GluR2-GRIP1 interaction but not GluR2-Pick1 binding [31]. Accordingly, it is more plausible that GluR2 Ser880 is dephosphorylated rather than being phosphorylated following BDNF treatment. In agreement, the current results do not show increased GluR2 phosphorylation even after 3 days of daily BDNF treatment (Fig. 6B). Future studies should characterize phosphatases that may regulate GluR2 interaction with its scaffolding proteins as well as kinases and phosphatases that can directly act on scaffolding proteins [60].

Even with recent progress, the full implications of BDNF-induced translocation of GluR2 and GluR2-containing AMPAr complexes to the surface of neurons are not well understood. Acute BDNF application acts rapidly to facilitate the induction of LTP and the short time interval within which GluR2 translocation occurs is highly suggestive that it contributes to LTP [27], [39]. In neurons, BDNF-mediated mutual upregulation of GluR2 and GRIP1 can have considerable implications for the effects of the neurotrophin to offset schizophrenia and behavioral depression and promote memory maintenance and synaptic potentiation during late phase LTP (l-LTP) [47], [48], [61], [62]. For instance, acute BDNF application drives GluR2 interaction with *N*-ethyl-maleimide sensitive factor (NSF) causing translocation of GluR2 and GluR2-containing AMPAr complexes to the cell surface in both primary neuronal cultures and HEKTrkB cells, independently from NMDA receptor activation and synaptic activity [27]. Although GluR2-NSF interaction is involved in synaptic enhancement during l-LTP and in memory maintenance, as it enhances trafficking of GluR2-containing AMPAr to the synapse, the mechanisms through which GluR2-containing AMPAr are retained at synaptic sites remain unclear [27], [46]. BDNF is essential for the maintenance of l-LTP and persistence of long term memory as interference with BDNF binding to TrkB receptors specifically abrogates LTP maintenance (l-LTP), reduces LTP induction, and brings potentiated synapses back to baseline activity levels [42]. Similarly, interference with GluR2 synaptic trafficking and maintenance at the cell membrane abrogates memory and eliminates l-LTP [14], [15], [46].

Acute BDNF treatment can activate Calcium/Calmodulin-dependent Protein Kinase II (CaMKII) and stimulate CaMKII translation [50], [63], [64]. In addition, CaMKII phosphorylates SAP97 and drives it into dendritic spines [65]. It is therefore conceivable that the BDNF-mediated upregulation of SAP97 protein levels and interaction with GluR1 involves CaMKII leading to upregulation of GluR1 at neuronal cell surface. Indeed, BDNF treatment has recently been found to induce GluR1 translocation to the cell membrane and insertion at synaptic sites [53]. In agreement with the known role of BDNF in LTP, our results show that BDNF treatment of cultured neurons increases the accumulation of AMPAr subunits at the cell membrane (Fig. 2). Considering the reduced levels of BDNF in behavioral depression and the recent implication of AMPAr activation in antidepressant treatments, future studies should examine the role of BDNF on GluR1-SAP97 and GluR2-GRIP1 interactions and upregulation of both categories of proteins in the contexts of behavioral depression and antidepressant treatment [66]–[69].

It is important to note that the GluR1-SAP97 interactions demonstrated in our results only apparently disagree with previously published data where little GluR1-SAP97 interaction is detected in co-immunoprecipitation experiments; the vehicle-treated condition in our results (Fig. 2) shows little GluR1-SAP97 interaction exactly as in the co-immunoprecipitation data from biochemically-fractionated adult rat hippocampal synaptic membrane preparations [35]. Indeed, only the BDNF-treated samples have significantly enhanced GluR1-SAP97 interaction (Fig. 2). In addition, the difference could be due to two additional factors, embryonic primary cortical neuronal cultures are used instead of adult hippocampal protein extracts, and the detergent used in our experiments is deoxycholate and not TritonX-100 or SDS [31], [70], [71]. Various isoforms of SAP97, GRIP1 and ABP/GRIP2 have been cloned and these isoforms are enriched in distinct subcellular domains where they have been implicated in trafficking and/or anchoring AMPAr subunits at synaptic and intracellular sites [2]. Our previous results show that the density of SAP97 immunostaining that is adjacent to two presynapctic markers, synaptobrevin and synaptophysin, is increased following BDNF treatment of cultured cortical neurons [31]. In addition, at least two other studies indicate that alternatively-spliced isoforms of SAP97 are localized within distinct domains of dendritic spines, where they influence not only the cell-surface accumulation and exchange kinetics of GluR1-containing AMPA receptors, but also the access of these receptors to synaptic glutamate [71], [72].

SAP97 and GRIP1 are substrates for calpain, a Ca^2+^-dependent protease involved in NMDA-dependent induction of LTP [32], [70], [73]. Earlier studies proposed that GluR2-GRIP1 binding stabilizes a cytoplasmic pool of AMPAr subunits and this has been linked with NMDA-dependent activation of calpain, which causes GRIP1 degradation and release of GluR2 from its interaction with GRIP1 to promote exocytosis and cell membrane insertion of AMPA receptors [73], [74]. BDNF treatment drives GluR1 to synapses and GluR1-SAP97 interaction is also suggested to participate in GluR1 trafficking to synaptic sites [53], [55]. BDNF treatment activates the protease calpain in neurons [75]. As such, BDNF-mediated activation of calpain can possibly cause degradation of SAP97 and GRIP1. Calpain activation following BDNF treatment and synaptic activity degrades ankyrin repeat-rich membrane-spanning/kinase D-interacting substrate of 220 kDa (ARMS/Kidins220) and releases GluR1 from its binding to ARMS/Kidins220 [76], [77]. Although SAP97 and GRIP1 are substrates of calpain, which can be rapidly activated by BDNF, the current and previous results do not display BDNF-mediated reduction in native SAP97 and GRIP1 protein levels in HEKTrkB cells, whether expressed individually or together with GluR1, GluR2 and GluR2Δ5. Instead, prolonged BDNF treatment increases SAP97 and GRIP1 protein levels rather than leading to their calpain-dependent degradation [31]. In addition, the current results argue against a role for BDNF in increasing AMPAr accumulation in the cytoplasmic fraction as BDNF treatment increases AMPAr subunits in the cell membrane fraction and at the surface of BDNF-treated neurons as evidenced with the biotinylation results (Fig. 1 and Fig. 2). Accordingly, BDNF-mediated stabilization/accumulation of AMPAr subunits is better associated with scaffolding proteins interacting with membrane-bound AMPAr subunits rather than with AMPAr subunits found in the cytoplasmic pool. As GluR1, GluR2, SAP97 and GRIP1 are all substrates of calpain future studies should address the relationships between BDNF-dependent activation of calpain and their susceptibility to degradation by calpain following BDNF treatment; namely, whether phosphorylation of all of these proteins alters their susceptibility to degradation by calpain. Future studies will also be directed at determining whether various isoforms of these scaffolding proteins are differentially affected by BDNF treatment and calpain activation [60].

Taken together, the current results strongly support a mutual stabilization model for AMPAr subunits and their scaffolding proteins and provide new insights on acute mechanisms induced with BDNF treatment leading to specific upregulation of GluR2-GRIP1 interaction and to prolonged increases of their total protein levels. The results also imply tight co-regulation of each AMPAr subunit and its scaffolding protein. Accordingly, we propose that, despite being subjects to distinct regulatory mechanisms, AMPAr-PDZ protein interactions are essential for BDNF-mediated upregulation of GluR1, GluR2, SAP97 and GRIP1.

In conclusion, the current findings shed new lights on the interactions of AMPAr subunits with their scaffolding proteins under physiological conditions. The current findings can explicate previously unknown aspects of the role of BDNF in the perturbed systems associated with brain diseases and neuropsychiatric disorders that exhibit altered BDNF signaling and reduced AMPAr levels.

## Supporting Information

Figure S1 **Illustration of the analysis method used to quantify protein knock-down by immunostaining.** HEK293 cells were transduced with GAPDH dsRNA oligonucleotides (50 nM) to reduce endogenous GAPDH protein expression. 48 h after dsRNA transduction cells were fixed and stained with anti-GAPDH antibody (Millipore). Illustration of images corresponding to steps of analysis (A), analyzed image parameters (B) and corresponding bar-graphs (C) generated by a custom-made image-processing program; only total particle area, bolded in (B), was used for statistical result analysis.
(TIF)

Figure S2 **Knock-down of endogenous GAPDH and exogenously-expressed GluR1 in HEK293 cells.** GAPDH-specific dsRNA and GluR1-specific dsRNA oligonucleotides were transduced into GluR1-expressing HEK293 cells. A) Low magnification photomicrographs showing examples of staining of GluR1-transfected HEK293 with GAPDH and GluR1 C-terminal antibodies. B) Quantitation of immunostaining was done as in Figure S1. Results indicated significant reduction of GluR1-like and GAPDH-like labeling in cells transduced with the indicated oligonucleotides, with the most significant results obtained with the utilization of dsRNA #2 and #4 in combination. *, *p*<0.05; **, *p*<0.01 (n = 6).
(TIF)

Figure S3 **AMPAr subunit expression following knock-down of endogenous GluR1.** A) Immunostaining with anti-C-terminal GluR1 antibody 72 h after transducing DIV 10 cultured cortical neurons with the indicated concentrations of dsRNA oligonucleotides #2 and #4. B) Immunostaining with anti-C-terminal GluR1 or anti-C-terminal GluR2/3 antibodies 72 h after transducing DIV 10 cultured cortical neurons with equal amounts of dsRNA oligonucleotides #2 and #4 (70 nM final concentration). GluR1-specific dsRNA utilization resulted in specific reduction of GluR1-like labeling without showing visible reduction in GluR2-like staining.
(TIF)

Figure S4 **Model summarizing differences between GluR1-SAP97 and GluR2-GRIP1 interactions.** A) Previous results from heterologous co-expression of GluR1 and SAP97 in regular HEK293 cells show increased accumulation of both proteins when expressed together, well above the expression levels of each one of them when expressed individually [31]. B) GluR2-GRIP1 co-expression, however, does not lead to increased accumulation of these proteins as their levels are comparable to when individually expressed [31]. C, D) Schematic representation of results shown in Fig 6; BDNF treatment for up to 3 days of HEKTrkB cells expressing GluR2 or GRIP1 alone does not lead to their individual accumulation. However, acute BDNF treatment of HEKTrkB cells co-expressing GluR2 and GRIP1 increases their interaction (Fig. 6A). In addition, prolonged BDNF treatment (for up to 3 days) causes accumulation of co-expressed wild type GluR2 and GRIP1 but not GluR2Δ5 or GRIP1.
(TIF)

Supporting Information S1 **Supporting Materials and Methods and Supplemental Results.**
(DOCX)
