# Supplementary figures and images for: Acute BDNF Treatment Upregulates GluR1-SAP97 and GluR2-GRIP1 Interactions: Implications for Sustained AMPA Receptor Expression
Source: PLoS One. 2013 Feb 27;8(2):e57124. doi: 10.1371/journal.pone.0057124 (PMC3584105; doi:10.1371/journal.pone.0057124)

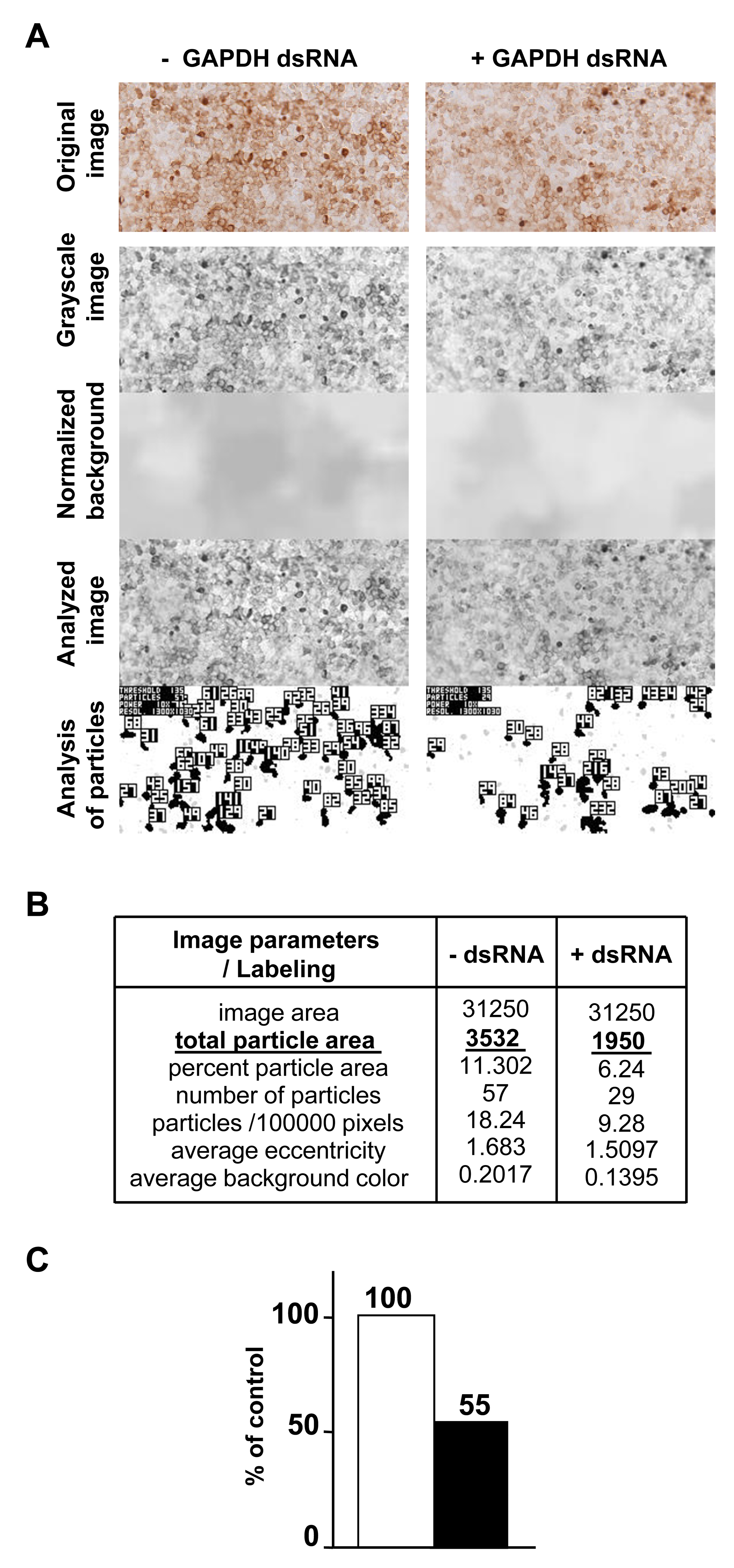

Supplement: Figure S1 — Illustration of the analysis method used to quantify protein knock-down by immunostaining. HEK293 cells were transduced with GAPDH dsRNA oligonucleotides (50 nM) to reduce endogenous GAPDH protein expression. 48 h after dsRNA transduction cells were fixed and stained with anti-GAPDH antibody (Millipore). Illustration of images corresponding to steps of analysis (A), analyzed image parameters (B) and corresponding bar-graphs (C) generated by a custom-made image-processing program; only total particle area, bolded in (B), was used for statistical result analysis. (TIF) [file pone.0057124.s001.tif]

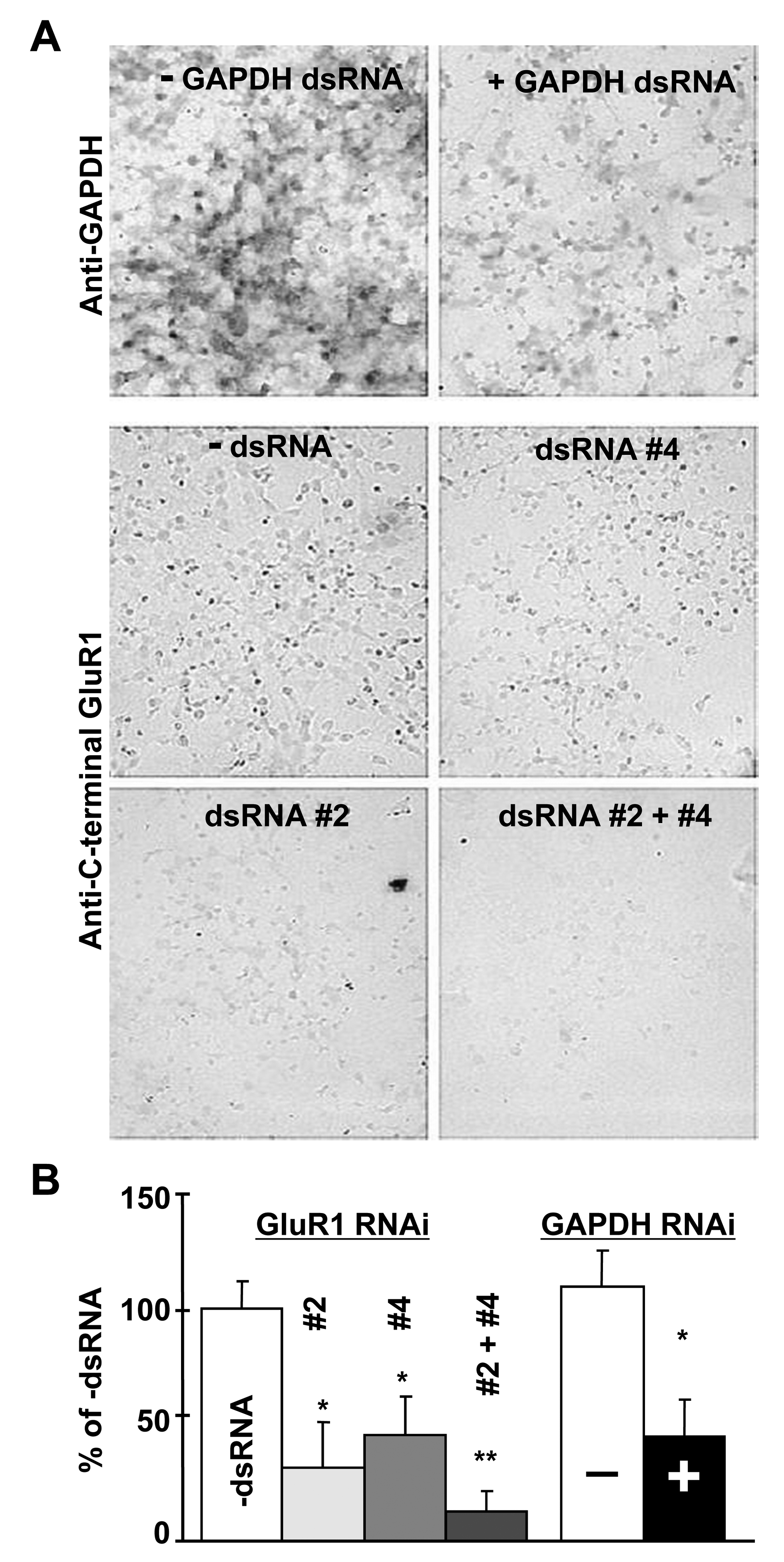

Supplement: Figure S2 — Knock-down of endogenous GAPDH and exogenously-expressed GluR1 in HEK293 cells. GAPDH-specific dsRNA and GluR1-specific dsRNA oligonucleotides were transduced into GluR1-expressing HEK293 cells. A) Low magnification photomicrographs showing examples of staining of GluR1-transfected HEK293 with GAPDH and GluR1 C-terminal antibodies. B) Quantitation of immunostaining was done as in Figure S1. Results indicated significant reduction of GluR1-like and GAPDH-like labeling in cells transduced with the indicated oligonucleotides, with the most significant results obtained with the utilization of dsRNA #2 and #4 in combination. *, p<0.05; **, p<0.01 (n = 6). (TIF) [file pone.0057124.s002.tif]

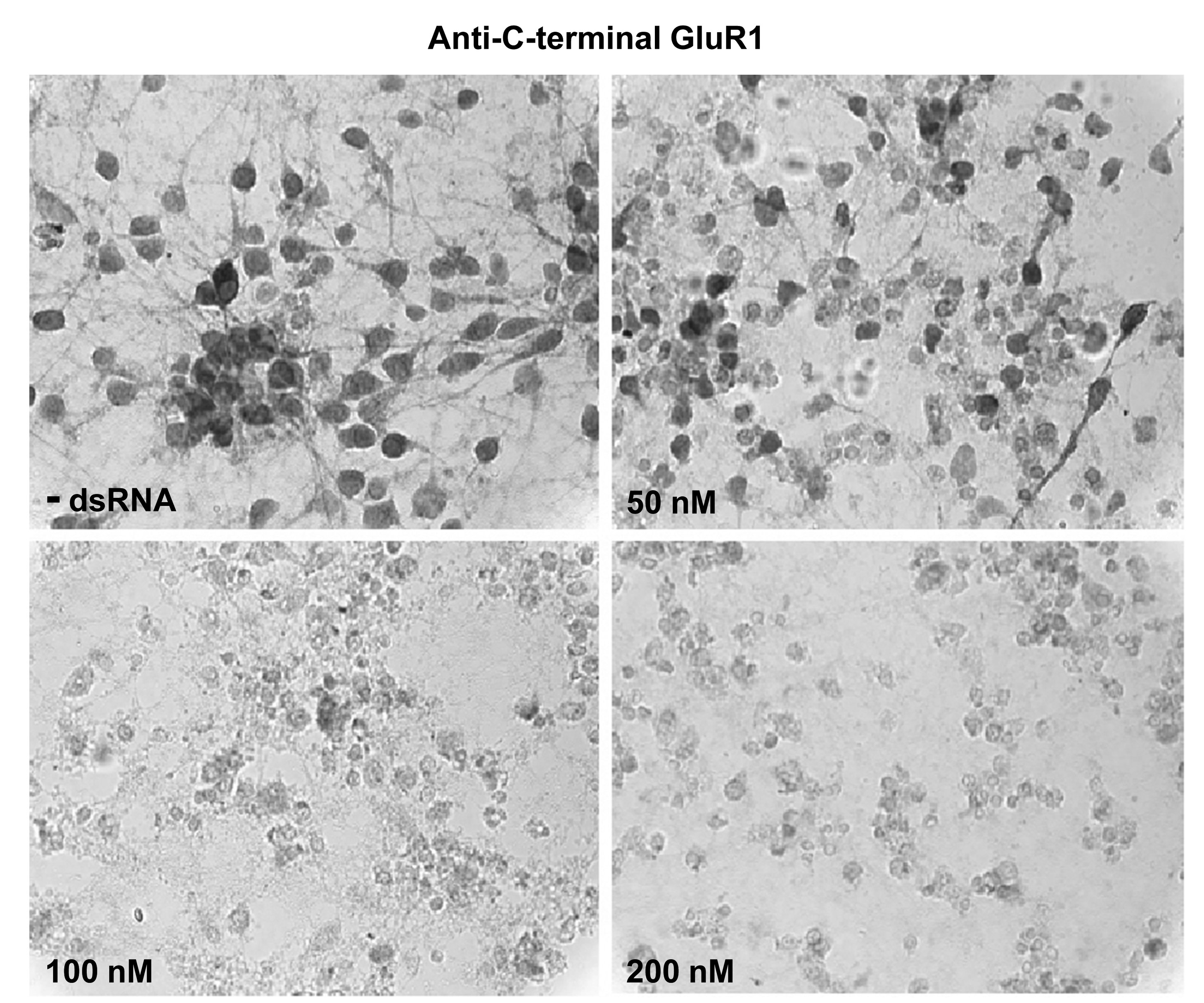

Supplement: Figure S3 — AMPAr subunit expression following knock-down of endogenous GluR1. A) Immunostaining with anti-C-terminal GluR1 antibody 72 h after transducing DIV 10 cultured cortical neurons with the indicated concentrations of dsRNA oligonucleotides #2 and #4. B) Immunostaining with anti-C-terminal GluR1 or anti-C-terminal GluR2/3 antibodies 72 h after transducing DIV 10 cultured cortical neurons with equal amounts of dsRNA oligonucleotides #2 and #4 (70 nM final concentration). GluR1-specific dsRNA utilization resulted in specific reduction of GluR1-like labeling without showing visible reduction in GluR2-like staining. (TIF) [file pone.0057124.s003.tif]

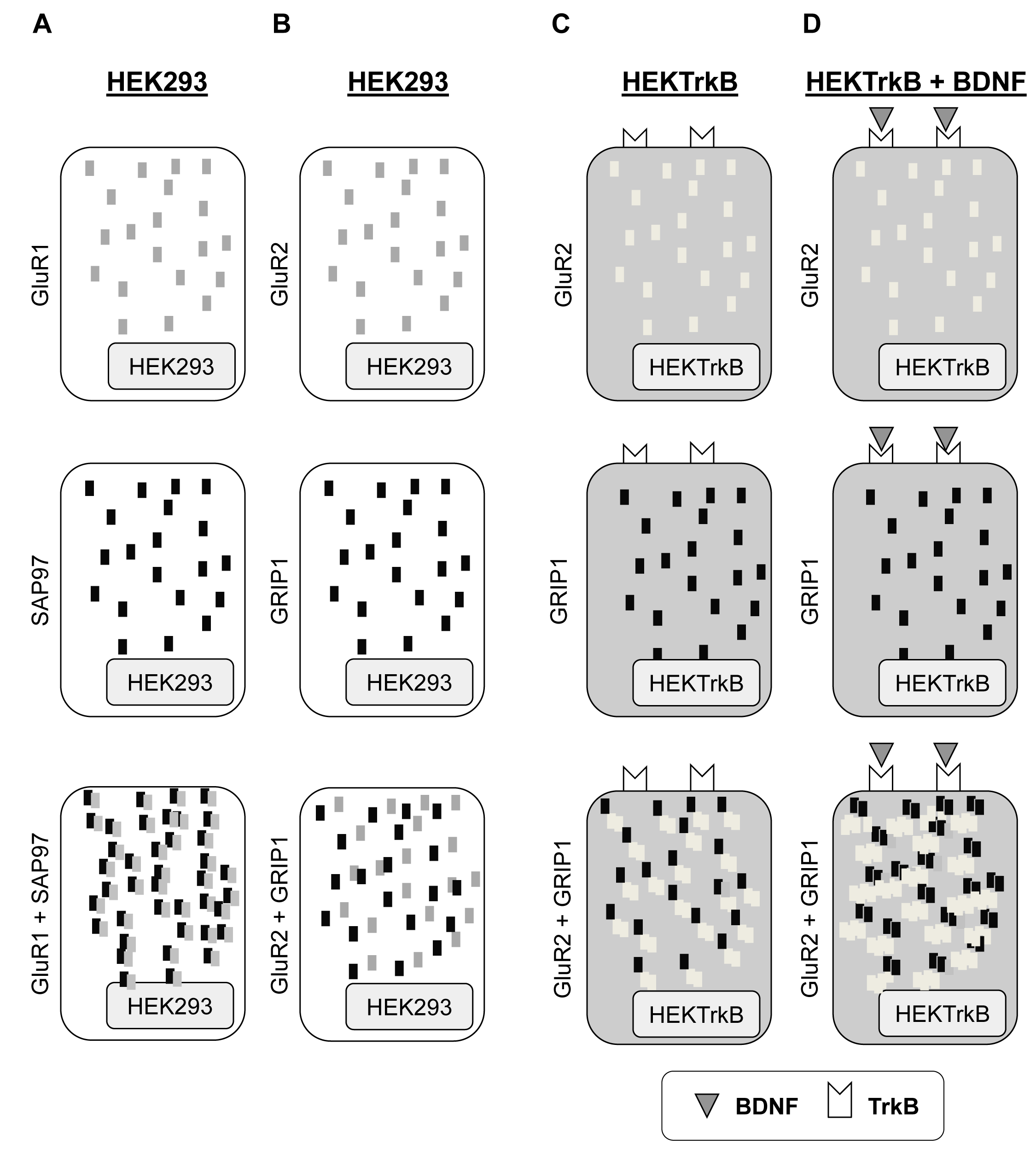

Supplement: Figure S4 — Model summarizing differences between GluR1-SAP97 and GluR2-GRIP1 interactions. A) Previous results from heterologous co-expression of GluR1 and SAP97 in regular HEK293 cells show increased accumulation of both proteins when expressed together, well above the expression levels of each one of them when expressed individually [31]. B) GluR2-GRIP1 co-expression, however, does not lead to increased accumulation of these proteins as their levels are comparable to when individually expressed [31]. C, D) Schematic representation of results shown in Fig 6; BDNF treatment for up to 3 days of HEKTrkB cells expressing GluR2 or GRIP1 alone does not lead to their individual accumulation. However, acute BDNF treatment of HEKTrkB cells co-expressing GluR2 and GRIP1 increases their interaction (Fig. 6A). In addition, prolonged BDNF treatment (for up to 3 days) causes accumulation of co-expressed wild type GluR2 and GRIP1 but not GluR2Δ5 or GRIP1. (TIF) [file pone.0057124.s004.tif]
