# Supplementary material for: Acute BDNF Treatment Upregulates GluR1-SAP97 and GluR2-GRIP1 Interactions: Implications for Sustained AMPA Receptor Expression
Source: PLoS One. 2013 Feb 27;8(2):e57124. doi: 10.1371/journal.pone.0057124 (PMC3584105; doi:10.1371/journal.pone.0057124)
Supplement: Supporting Information S1 — Supporting Materials and Methods and Supplemental Results. (DOCX) [file pone.0057124.s005.docx]

**MATERIALS AND METHODS**

**RNA interference in HEK293 cells and cultured cortical neurons**

HEK293 cells were used in preliminary RNAi experiments to determine the efficacy of transduced GAPDH- or GluR1-specific double stranded RNA nucleotides (dsRNA) in knocking-down the expression of endogenous GAPDH or exogenously-expressed GluR1. HEK293 cells were plated at 60-80% confluence and transfected 24 h later with expression vector carrying GluR1 cDNA using FuGene6 (Roche) [1]. The next day transfected cells were re-suspended and plated onto coverslips or chamber slides. Varying concentrations (0, 20, 50, 70, 100, and 200 nM) of dsRNA oligonucleotides were used in preliminary experiments (data not shown) to optimize GluR1 knock-down conditions in HEK293 cells and determine the most efficient dsRNA oligonucleotides. RNA (21 nucleotides in length) sequences were designed, chemically synthesized, annealed to make dsRNA according to the manufacturer’s recommendations (Ambion) and introduced into GluR1-transfected HEK293 cells by lipofection with FuGene6 or Lipofectamine 2000 according to the manufacturers’ recommendations (Roche and Invitrogen, respectively). Statistically significant reduction of exogenously-expressed GluR1 protein was observed at 70 nM; this concentration was used for all remaining experiments. Transfection of primary neuronal cultures was carried out according to published protocols [2,3]; different concentrations of GluR1-specific dsRNA (0, 50, 100, 200 nM) were formulated with TransMessenger transfection reagent and condensed with Enhanser R as recommended by the manufacturer (Qiagen) and used in preliminary experiments with cultured neurons to knock-down endogenous GluR1 protein (Figure S3). The dsRNA-reagent complex was diluted in 900 μl of Neurobasal medium supplemented with B27 and 0.5 mM glutamine, added directly to DIV 10 neuronal cultures and replaced 2 h later with B27-Neurobasal medium [3]. Additional experiments determined that 70 nM was the lowest GluR1-specific dsRNA concentration that resulted in reproducible and statistically-significant reduction of endogenous GluR1 expression; this concentration was used in the remaining experiments (Figure S3). HEK293 cells and primary neurons were stained 48–72 h after transfections, fixed (4% paraformaldehyde in 0.1 M phosphate buffer, pH 7.4), and incubated with anti-GAPDH antibody (Millipore) or anti-C-terminal GluR1 antibody (Millipore). Immunoreactivities were revealed using biotin-conjugated secondary antibodies, the ABC kit (Vector Laboratories) combined with the diaminobenzidine method, and visualized with the aid of a Zeiss microscope (Axioskop) fitted with an LCD camera (DP50-CU; Olympus). In addition, total protein samples from similarly-treated neuronal cultures were processed for immunoblotting as indicated in “Materials and Methods” and the results are shown in Fig. 4. The GluR1-specific dsRNA oligonucleotides corresponded to the cDNA of rat GluR1 protein (GenBank Accession Number: 1602240A; Reference Sequence: NM_031608.1): 5'-aatcacaggaacatgcggctttt-3' (#1), 5'-aaaaggagaggctggtggtggtt-3' (#2), 5'-aaagcctgcggaggcagaggatt-3' (#3), and 5’-gaagtctgcagaaccatccgtgtt-3' (#4).

**Quantitation of GluR1 knock-down and image processing**

Pictures of GAPDH- and GluR1-stained cells were taken with a 20x objective, at 1/300 s shutter speed using Studio Lite software (Pixera Corp., CA). Four images per condition were analyzed and results were obtained from 2 experiments, each with 3 similar cultures per condition (n=6). All pictures were fed into and analyzed using costume-made software (Dr. G. Lynch, University of California at Irvine). This image processing program, a console application custom-designed in a mix of Perl and C programming languages, converted images from color to 8 bit per pixel grayscale, saved them in “.pme” format, estimated the local background, and normalized the pixel values so that the background is a fixed level of gray. A visually-set threshold of background immunostaining was used for all pictured fields. This normalization was necessary because cell and background staining varied across image fields. The normalized image was then converted to a binary image according to user-provided arbitrary threshold value that was kept constant for all conditions of each experiment. The resulting binary image was then cleaned by 'dilation' and 'erosion' to remove stray pixels and close small gaps. Groups of connected pixels (particles) were then detected and their statistical properties analyzed. Particles too elongated (labeled fibers rather than cell bodies) were rejected and excluded from calculation using a fixed threshold on eccentricity. While no measurements were calculated for these rejected particles, they were still displayed in the program image output as a quality control feature. Particles too small were rejected using a fixed threshold on area. This program calculated the total surface area of immunostaining above the visually-set threshold, which was the same for all analyzed photos, and generated an Excel (Microsoft, Palo Alto, CA) -readable file comprising various other measurements and statistics of the captured image. Results and global statistics (i.e. nonparametric, one and two-tailed *t*-tests) were then collected, saved and used to generate finalized data. *P* values < 0.05 were considered to indicate statistically significant differences between experimental conditions. Figure S1 shows an example of the result analysis and output from one photographed field per condition.

**RESULTS**

**Knock-down of heterologous GluR1 protein expression in HEK293 cells**

GluR1-expressing HEK293 cells were transduced with GluR1- and GAPDH-specific dsRNA oligonucleotides as indicated in earlier. The GAPDH-specific oligonucleotides significantly reduced endogenous GAPDH expression (42 ± 18%; *p* < 0.05) as compared to control cultures (Figure S1 & Figure S2). High protein levels of exogenously-expressed GluR1 protein were detected in HEK293 cells by immunocytochemistry (Figure S2). For GluR1 the knock-down efficiency of 4 dsRNA sequences used individually or in combinations was examined as indicated earlier. In parallel, GAPDH-specific dsRNA was also used as positive control to knock-down the expression of endogenous GAPDH (Figure S2). The levels of GluR1 and GAPDH expression were estimated with immunostaining, which was carried out as indicated in “*Supporting Information*”. The results indicated that oligonucleotides #2 and #4 (70 nM final concentration) were most effective when used in combination and resulted in reproducible and statistically-significant knock-down of GluR1, though each could still significantly reduce GluR1 protein expression when used individually (Figure S2). Oligonucleotides #1 and #3 were less efficient in all possible combinations (data not shown). In addition, GluR1 expression levels were reduced in HEK293 cells transfected with GluR1-specific dsRNA without affecting endogenous GAPDH expression levels (data not shown). The results were reproducible in 2 experiments, each with 3 sister cultures (n=6).

**GluR1 knock-down in cultured neurons**

Oligonucleotides #2 and #4 were next used in combination at increasing concentrations (0, 50, 100, and 200 nM) to reduce neuronal expression of GluR1 (Figure S3A). The results indicated that at 50 nM these oligonucleotides reduced endogenous GluR1 protein levels. At 100 and 200 nM, GluR1 staining showed highly-significant reduction in labeling. However, this was associated with the presence of neuron-like profiles with diffuse staining, suggesting incidence of neuronal death in discrete areas of the transduced cultures. Further experiments determined that combining oligonucleotides #2 and #4 (70 nM, final concentration) resulted in a reproducible reduction of GluR1-like labeling without diffuse staining. Thus, oligonucleotides #2 and #4 were used together at 70 nM to reduce endogenous GluR1 expression and examine its effects on the GluR1-scaffolding protein SAP97 and on the related AMPAr subunit GluR2 (Fig. 4). GluR1-like labeling of cultured neurons showed reduced staining in dsRNA-transduced neurons compared to mock-transduced cells without visible differences in GluR2-like staining (Figure S3B). Immunoblotting results from similarly-treated cultures are shown in Fig. 4. Interestingly, the reduction of GluR1 immunoreactivity was consistent with the rate of GluR1 protein turnover (41 h, 40 % turnover) [4].

**References:**

1. Jourdi H, Iwakura Y, Narisawa-Saito M, Ibaraki K, Xiong H, et al. (2003) Brain-derived neurotrophic factor signal enhances and maintains the expression of AMPA receptor-associated PDZ proteins in developing cortical neurons. Dev Biol 263(2): 216-230.
2. Gaudilliere B, Shi Y, Bonni A (2002) RNA interference reveals a requirement for myocyte enhancer factor 2A in activity-dependent neuronal survival. J Biol Chem 277(48): 46442-46446.
3. Krichevsky AM, Kosik KS (2002) RNAi functions in cultured mammalian neurons. Proc Natl Acad Sci U S A 99(18): 11926-11929.
4. Horikawa HP, Nawa H (1998) Turnover rates of the AMPA-type glutamate receptor GluR1 measured by transient gene expression. J Neurosci Methods 84(1-2): 173-179.
